# Value of serum GGT level in the timing of diagnosis of choledochal cyst perforation

**DOI:** 10.3389/fped.2022.921853

**Published:** 2022-08-15

**Authors:** Shuhao Zhang, Duote Cai, Qingjiang Chen, Yuebin Zhang, Ken Chen, Yi Jin, Wenjuan Luo, Zongwei Huang, Di Hu, Zhigang Gao

**Affiliations:** Department of General Surgery, The Children's Hospital, Zhejiang University School of Medicine, National Clinical Research Center for Child Health, Hangzhou, China

**Keywords:** choledochal cyst, perforation, cystic necrotic changes, gamma-glutamyl transpeptidase, prediction

## Abstract

**Background:**

Choledochal cyst perforation is extremely rare, and early diagnosis or prediction is important for the immediate therapeutic intervention of perforations. This study aimed to define the predictor(s) of an impending or complete spontaneous perforation of choledochal cyst and establish the optimal operative timing.

**Methods:**

All 429 consecutive choledochal cyst patients from January 2015 to December 2021, were included. A retrospective study was performed based on Kaplan-Meier analysis, and Cox univariate and multivariate analyses.

**Results:**

A total of 429 patients were included, among which, 21 had choledochal cyst perforations (group A), and 408 did not (group B). Compared to group B, the serum alanine aminotransferase, aspartate aminotransferase, direct bilirubin, gamma-glutamyl transpeptidase, indirect bilirubin, total bilirubin, and alkaline phosphatase were significantly higher in group A (*p* = 0.025, 0.006, < 0.0001, 0.0001, 0.001, < 0.0001, and 0.033). High serum gamma-glutamyl transpeptidase was negatively associated with perforation-free preoperative survival, and multivariate Cox regression revealed that serum gamma-glutamyl transpeptidase was an independent predictive factor for an impending or complete perforation (*p* = 0.042).

**Conclusions:**

A gamma-glutamyl transpeptidase level ≥ 346.5 U/L accompanied with significantly elevated liver enzymes and bilirubin levels was indicative of the possibility of an impending or complete choledochal cyst perforation, and a proactive surgical approach should be considered.

## Introduction

Choledochal cysts (CCs) are cystic or fusiform dilations of the common bile duct. CCs occur in one in 1,000 persons in Asia, while they are less frequent, at one in 50,000–150,000 individuals, in the West. The female-to-male ratio for CCs ranges from 3:1 to 4:1 (1, 2). CC perforation is extremely rare, with a reported frequency of 1.8–2.8% (3, 4). The pathogenesis of spontaneous perforation has not been clarified, but proposed mechanisms include the reflux of pancreatic secretions (5), increased intraluminal pressure due to protein plugs (6), and viral infection (7).

CCs are treated by elective surgery, and patients usually experience a satisfiable prognosis in most cases. Whereas, as an acute abdomen, CC perforation requires emergency surgery, a delay in the diagnosis or inappropriate management can result in the development of complications (bacterial contamination of the biliary ascites, life-threatening sepsis, attacks of cholangitis, and pancreatitis) (8), which will prolong the course, increase the cost of hospitalization, and exacerbate the pain of patients.

Therefore, early diagnosis or prediction is important for the immediate therapeutic intervention of impending or complete CC perforations. As a specialized children's hospital and national clinical research center, we have performed surgeries on more than 600 CCs since January 2011. Here, we conducted a retrospective study to summarize the features of patients with CC perforations and explored the potential predictive factors that could be used to contribute to the timely diagnosis and management of CC perforations.

## Materials and methods

### Study design and patients

Following institutional review board approval, the electronic medical records of consecutive patients (104 males and 325 females) who underwent excision of CCs and Roux-en-Y hepaticojejunostomy in the Department of General Surgery, Children's Hospital of Zhejiang University School of Medicine between January 2015 and December 2021 were collected. Twenty-one patients (six males and 15 females) were complicated with CC perforations, as confirmed by intraoperative examinations. All operations were performed by the same group of experienced surgeons. Variables, including patient demographics, symptoms, classification of cystic or fusiform dilatation, and serum biochemical indices were recorded. A retrospective analysis was performed between patients with or without CC perforation, based on the above clinical data.

### Inclusion criteria

The inclusion criteria were: (1) patients diagnosed with CC based on the intraoperative findings and postoperative pathological results; (2) the Children's Hospital of Zhejiang University School of Medicine was the first-visit hospital for patients; (3) patients did not have any other abdominal surgeries; and (4) CC perforation or cystic necrotic changes were verified by intraoperative examinations. CC patients who did not meet the above four criteria were excluded.

### Classification of perforation type and perforation locations

Type I: complete cystic perforation; type II: sealed perforation with a small amount of bile contained in a pseudocyst; and type III: cystic necrotic changes with an intact serosal layer. Perforation locations of choledochal cyst were shown in Table 1.

**Table 1:** Perforation location of choledochal cyst.

**Perforation location**	**Number**
Posterior wall	8
Anterior wall	6
Near the neck of gallbladder	4
Multiple perforations (including anterior and posterior wall)	3

### Statistical analysis

Data are shown as the medians ± interquartile ranges (IQRs). Statistical analyses were performed using SPSS22.0 and GraphPad Prism 6. Statistical differences were analyzed by two-tailed Student's *t*-tests. Survival curves were drawn using Kaplan–Meier univariate estimates according to the Youden index. Multivariate analysis was performed by Cox regression. A *p*-value < 0.05 was considered statistically significant.

## Result

### Clinical data and analyses of serum biochemical indices

A total of 429 CC patients (104 males and 325 females) were included in this study, among whom 21 (six males and 15 females) were complicated with perforation (group A), and 409 (98 males and 310 females) did not experience perforation (group B). The two groups of patients were similar in sex, and age. The predominant symptoms were abdominal pain (61.9% in group A and 57.6% in group B) and vomiting (71.4% in group A and 46.5% in group B), followed by jaundice, fever and acholic stool. Within group A, six patients (28.6%) had type I perforations, and 15 patients (71.4%) had type III perforations. Group A exhibited higher serum ALT levels [130.00 (54.00–226.00) vs. 40.50 (19.00–126.50), *p* = 0.025], and serum AST levels [111.00 (62.00–199.00) vs. 55.00 (34.25–127.75), *p* = 0.006] compared to group B. The serum levels of direct bilirubin [20.80 (4.45–49.20) vs. 3.00 (1.60–9.52), *p* < 0.0001], indirect bilirubin [23.25 (12.93–42.45) vs. 7.80 (4.50–17.50), *p* = 0.001], total bilirubin [47.00 (18.93–102.43) vs. 10.40 (6.30–29.10), *p* < 0.0001], and alkaline phosphatase [468.00 (256.50–754.50) vs. 10.40 (6.30–29.10), *p* = 0.033] in group A were significantly higher than those in group B. Significantly elevated serum GGT levels [478.00 (213.00–604.00) vs. 123.00 (25.00–376.00), *p* = 0.0001] were also observed in group A compared to group B (data are shown in Table 2).

**Table 2:** Demographic and laboratory data of CC patients with or without perforation.

	**CC perforation**	**CC without perforation**	***p***
Sex (male: female)	6:15	98: 310	*X*^2^= 0.225 *p =* 0.635
Age (month)	20.00 (11.00–40.00)	28.50 (8.25–52.75)	*p =* 0.181
**Symptom**			
Abdominal pain	13	235	X^2^= 0.152 *p =* 0.697
Fever	3	31	*p =* 0.227
Jaundice	5	42	*p =* 0.067
Acholic stool	4	26	*p =* 0.050
Vomiting	15	190	*X*^2^= 9.838 *p =* 0.002
Asymptomatic	2	119	*X*^2^= 3.805 *p =* 0.051
**Perforation**			
Type I	6	/	/
Type III	15	/	/
Albumin (normal range: 32–52g/L)	42.00 (38.20–44.05)	43.20 (40.60–46.20)	0.1
Alanine aminotransferase (normal range: 0–50 U/L)	130.00 (54.00–226.00)	40.50 (19.00–126.50)	**0.025**
Amylase (normal range: 40–132 U/L)	73.50 (32.40–150.15)	62.40 (31.55–112.63)	0.461
Aspartate aminotransferase (normal range: 15–60 U/L)	111.00 (62.00–199.00)	55.00 (34.25–127.75)	**0.006**
Total cholesterol (normal range: 3–5.7 mmol/L)	4.92 (4.41–6.01)	4.50 (3.90–5.36)	0.085
Direct bilirubin (normal range: 0–5.1μmol/L)	20.80 (4.45–49.20)	3.00 (1.60–9.52)	**<0.0001**
Gamma-glutamyl transpeptidase (normal range: 8–57 U/L)	478.00 (213.00–604.00)	123.00 (25.00–376.00)	**0.0001**
Indirect bilirubin (normal range: 1–20 μmol/L)	23.25 (12.93–42.45)	7.80 (4.50–17.50)	**0.001**
Total bile acid (normal range: 0-13 μmol/L)	17.30 (3.55-73.10)	7.50 (3.50-18.08)	0.139
Total bilirubin (normal range: 5–21 μmol/L)	47.00 (18.93–102.43)	10.40 (6.30–29.10)	**<0.0001**
Alkaline phosphatase (normal range: 42–362 U/L)	468.00 (256.50–754.50)	293.00 (227.00–407.00)	**0.033**

*Bold values denote the p-values < 0.05*.

### ROC curve and Kaplan-Meier analysis

**Figure 1:**
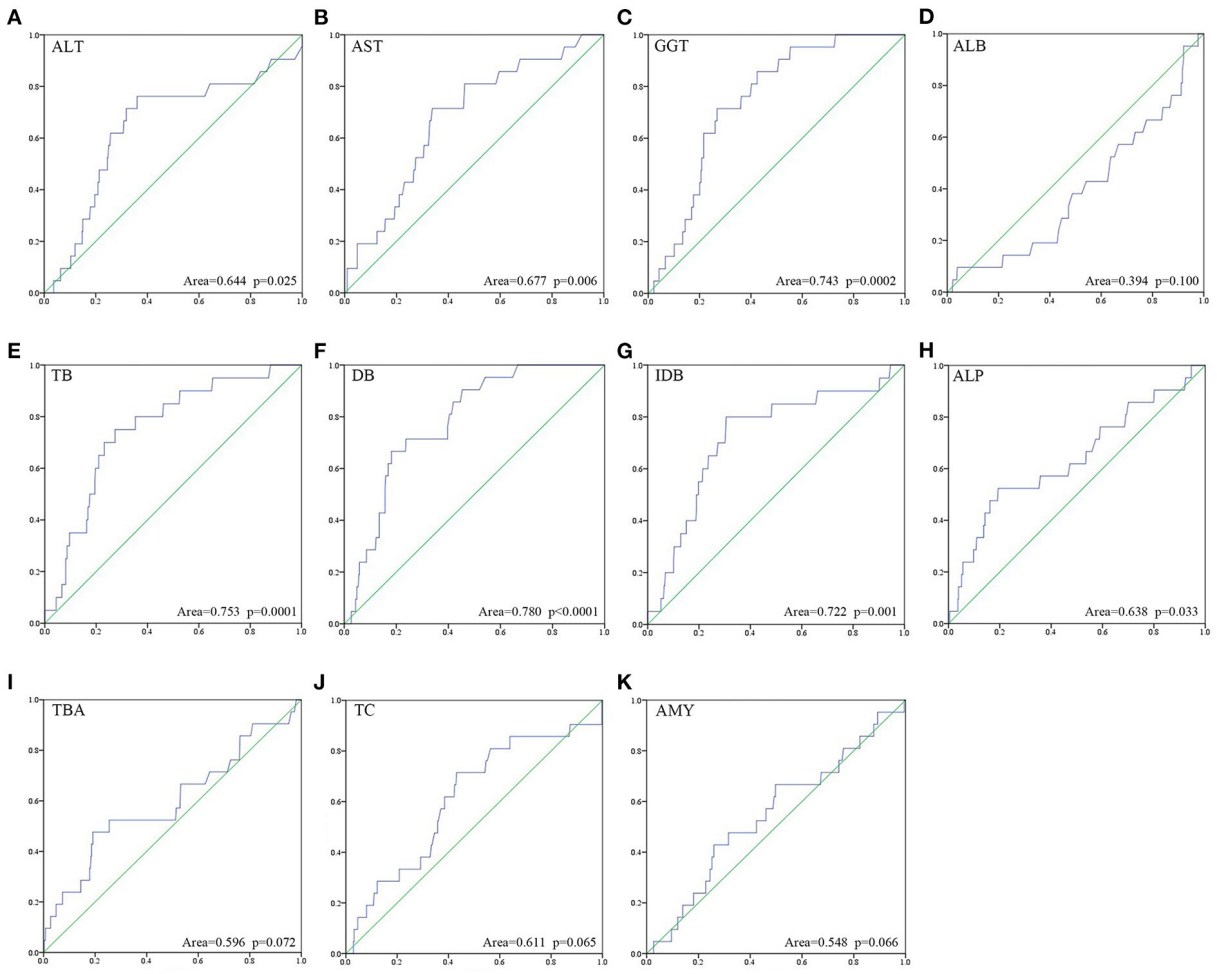
Univariate ROC curve analysis about risk of perforation onto each of the eleven serum biochemical indices **(A–K)** was performed.

**Figure 2:**
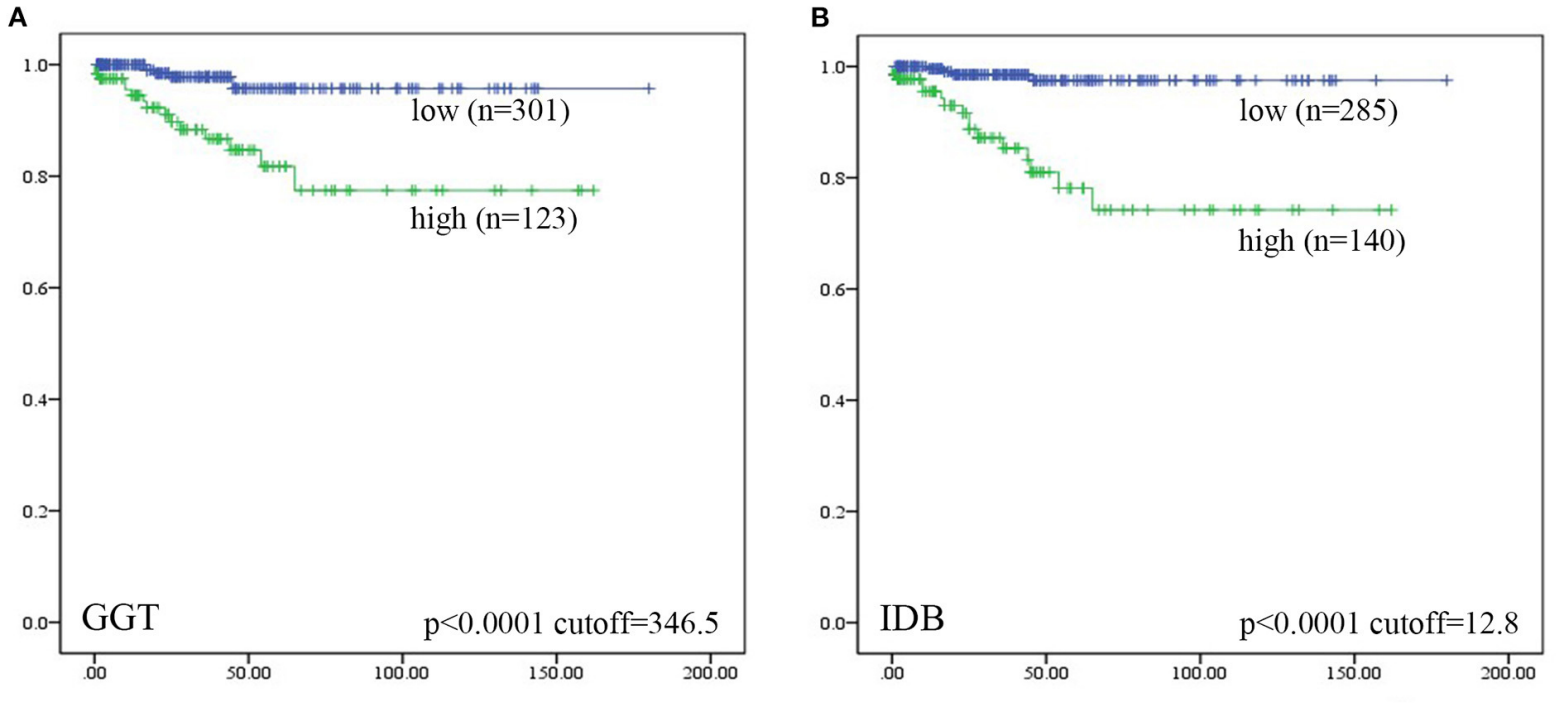
Patients were divided into “high” and “low” groups according to the optimal cut-offs which were determined by Youden index. Kaplan–Meier analyses of preoperative perforation-free survival for **(A)** serum GGT level, and **(B)** serum IDB level were analyzed. Among them, five patients lost data of GGT levels and four lost data of IDB levels. Log-rank test was used.

Univariate ROC curve analyses of the effect of CC perforation on each serum biochemical index was performed. The AUCs and *p*-values of ALT, AST, GGT, ALB, TB, DB, IDB, ALP, TBA, TC, and AMY are shown in Figure 1. The optimum cutoffs of serum GGT (cutoff = 346.5 U/L, sensitivity = 71.4%, specificity = 73.2%) and indirect bilirubin (cutoff = 12.8 μmol/L, sensitivity = 80.0%, specificity = 79.4%) levels were set according to the Youden index. Next, we evaluated the prognostic values of serum GGT (cutoff = 346.5 U/L) and indirect bilirubin (cutoff = 12.8 μmol/L) levels, and divided each into high and low groups according to the cut-offs. Using Kaplan–Meier curves, we identified that high levels of serum GGT and indirect bilirubin were negatively associated with preoperative perforation-free survival (*p*-values were both <0.0001, Figure 2).

### Cox univariate and multivariate analyses

To illustrate whether independent preoperative predictive factors existed, each serum biochemical index that was significant based on univariate analyses were adopted as covariates for multivariate Cox regression analyses. Patients with high serum GGT levels harbored a 1.001—fold higher risk of perforation than patients with low serum GGT levels (HR, 1.001; 95% CI, 1.000–1.002; *p* = 0.001). High serum levels of indirect bilirubin also imparted a 1.021—fold higher risk of perforation (HR, 1.021; 95% CI, 1.010–1.032; *p* = 0.021, Table 3). However, the cutoff level (12.8 μmol/L) of indirect bilirubin was within the normal range clinical testing (1–20 μmol/L in our hospital), and thus, we excluded indirect bilirubin and the clinical application of this index in predicting CC perforation remained further discussion.

**Table 3:** Cox univariate and multivariate analyses.

**Index**	**Univariate analyses**		**Multivariate analyses**	
	**HR (95%CI)**	***p***	**HR (95%CI)**	***p***
Sex		0.744		
Alanine aminotransferase		0.374		
Amylase		0.456		
Aspartate aminotransferase	1.003 (1.000–1.005)	**0.028**		0.682
Total cholesterol		0.466		
Direct bilirubin	1.018 (1.007–1.030)	**0.001**		0.611
Gamma-glutamyl transpeptidase	1.001 (1.001–1.002)	**0.001**	1.001 (1.000–1.002)	**0.042**
Indirect bilirubin	1.024 (1.013–1.034)	**<0.0001**	1.021 (1.010–1.032)	**0.0002**
Total bile acid	1.006 (1.001–1.012)	**0.011**		0.881
Total bilirubin	1.012 (1.007–1.018)	**<0.0001**		0.611
Alkaline phosphatase	1.001 (1.000–1.002)	**0.009**		0.616

*Bold values denote the p-values < 0.05*.

## Discussion

Choledochal cyst perforation is an uncommon complication, while bile peritonitis can be fatal (9). Because of their atypical clinical manifestations, preoperative misdiagnoses of CC perforation are high, and early pre-operative diagnoses are notoriously difficult to perform. Here, we aimed to summarize the clinical characteristics of patients with CC perforations and explore potential non-invasive serum biomarkers for the detection of perforations at early stages.

The exact pathogenesis of CC perforations remains controversial. Previous studies concluded three main potential causes of spontaneous perforation. First, the distal obstruction of the bile duct and consequent high intraluminal pressure may result in the perforation of the fragile cystic wall (10, 11). Thus, some researchers suggested that CC perforation is not rare in infancy because of the immature ducal wall with fewer elastic fibers (10, 12). Second, pancreaticobiliary malunion may cause epithelial irritation of the biliary tract due to refluxed pancreatic juice (10, 13, 14). Third, the poor blood supply of the common bile ducts' anterior wall, which is only from the marginal artery, is more susceptible to an ischemic event (15, 16). In addition to their undefined etiologies, CC perforations may also manifest clinically with atypical symptoms, such as acute abdomen with peritonitis and vomiting (10, 17). This was also observed in our series with abdominal pain (61.9%) and vomiting (71.4%) as the primary complaints. One patient with complete cystic perforation was even asymptomatic in our study. If the site of perforation is small, patients may present without any symptoms since the bile may escape slowly enough to permit a pseudocyst to develop (12). Thus, the early diagnosis of CC perforation is difficult and the frequencies of misdiagnoses are high. Not to mention cystic necrotic changes with an intact serosal layer, patients may not have any clinical symptoms with an impending perforation.

One previous study pointed out that the interruption of the continuity of the bile duct in ultrasound was a diagnostic feature for CC perforations, and exhibited a specificity of 100%. However, the detection sensitivity of the interruption on ultrasound was low (18.6%) (8). The loss of local gallbladder tension, a thickened gallbladder wall, abnormal morphology of the gallbladder, and peritoneal effusion could also be used as imaging features for CC perforation (18). Ultrasound allows for real-time dynamic monitoring and has good reproducibility, but it requires subjective assessments by the operator, which requires sufficient experience to become proficient. Thus, a non-invasive and objective serum biomarker is clinically superior. Serum GGT levels were used to predict biliary atresia, however, its accuracy varied (19, 20). Some studies also included GGT levels to develop a grading system (Grade I–III) for bile leakage severity in CC, with the serum GGT level for a Grade I diagnosis being 343.75 ± 215.6 U/L (6). In the current study, we found that preoperative serum GGT levels were positively correlated cystic necrotic change and complete CC perforation. Thus, we supported the early detection of elevated GGT level as an important reference for the possibility of CC perforation.

Serum GGT level was an independent predictive factor of CC perforation, and patients with low GGT levels (cutoff = 346.5U/L) experienced a long perforation-free preoperative survival duration. Collectively, we identified a potential non-invasive and objective biomarker to predict CC perforation. More importantly, we applied serum GGT level to predict potential cystic necrotic changes, which are indicative of an impending perforation, to help with timely clinical decision making. We also found that CC patients with cystic necrotic changes or complete perforation had significantly elevated levels of serum ALT, AST and bilirubin. Thus, those markers could be used as important auxiliary indicators to enhance the accuracy of clinical assessments. Thus, when encountering CC patients with GGT levels >346.5 U/L combined with significantly elevated liver enzymes and bilirubin, dynamic monitoring of ultrasound is recommended. We should especially focus on gallbladder fossa or right upper abdomen effusion indicated by ultrasound and finish MRCP examination as soon as possible. Then, a proactive surgical approach should be considered to avoid bile leakage, preferably before perforation.

## Conclusions

In summary, we identified the applicability of serum GGT levels in predicting CC perforation, particularly in patients with potential cystic necrotic changes, which were indicative of an impending perforation. Serum GGT level relied on routine clinical data, and thus, it is practical and easily accessible for clinical decision-making purposes. However, validation of its relationship with CC perforation is required in the future.

## Data availability statement

The original contributions presented in the study are included in the article/supplementary material, further inquiries can be directed to the corresponding author/s.

## Ethics statement

The studies involving human participants were reviewed and approved by the Ethics Committee of the Children's Hospital, Zhejiang University School of Medicine (Approval No. 2022-IRB-047). Written informed consent from the participants' legal guardian/next of kin was not required to participate in this study in accordance with the national legislation and the institutional requirements.

## Author contributions

SZ and DC contributed to study conception and design. SZ, DH, ZH, and QC contributed to data acquisition. SZ, YZ, KC, YJ, and WL contributed to analysis and data interpretation. SZ and ZG contributed to drafting of the manuscript, while ZG contributed to critical revision. All authors contributed to the article and approved the submitted version.

## Funding

This work was supported by the Clinical Medical Research of Minimally Invasive Diagnosis and Treatment of Abdominal Organs in Zhejiang Province (Grant No. 01492-02).

## Conflict of interest

The authors declare that the research was conducted in the absence of any commercial or financial relationships that could be construed as a potential conflict of interest.

## Publisher's note

All claims expressed in this article are solely those of the authors and do not necessarily represent those of their affiliated organizations, or those of the publisher, the editors and the reviewers. Any product that may be evaluated in this article, or claim that may be made by its manufacturer, is not guaranteed or endorsed by the publisher.
